# Focus on cutting‐edge research to improve the influence of *Animal Models and Experimental Medicine*

**DOI:** 10.1002/ame2.12210

**Published:** 2022-02-14

**Authors:** Chuan Qin

**Affiliations:** ^1^ Chinese Association for Laboratory Animal Sciences Beijing China; ^2^ Institute of Laboratory Animal Sciences, Chinese Academy of Medical Sciences & Peking Union Medical College Beijing China

The year 2022 marks the fifth year of the publication of *Animal Models and Experimental Medicine* (*AMEM*). We are intensely proud of the publication of each paper, because we recognize that all our efforts are worthwhile to provide a platform for communication and sharing of outstanding scientific results and reinforce the discipline position of animal models and experimental medicine.

*AMEM* is jointly published by the Chinese Society of Experimental Animals and the Institute of Medical Laboratory Animals of the Chinese Academy of Medical Sciences & Peking Union Medical College, in collaboration with Wiley, a renowned international publishing group. Since its inception in 2018, *AMEM* has received 333 manuscripts from 45 countries and regions, including Mainland China, the United States, India, Nigeria, Brazil, Bangladesh, Australia, Japan, Spain, and Israel. It has become an important international exchange platform for innovative research results in the fields of basic medicine and comparative medicine, and it has successively been included in domestic and foreign scientific databases such as Chinese Science Citation Database (CSCD), Directory of Open Access Journals (DOAJ), PubMed Central, and World Journal Clout Index (WJCI). In 2021, it was successively included in the MEDLINE and Clarivate Analytics Emerging Source Citation Index (ESCI) databases. Being included by ESCI signifies that *AMEM*'s academic level and international influence in the field of laboratory animal science have been acknowledged internationally and it has laid a solid foundation for the journal to become a world‐class journal.

At present, life sciences, especially the intersection and integration of medicine and engineering science, materials science, and information science are accelerating. The fields of experimental zoology and comparative medicine covered by *AMEM* are an important part of this integration process. It has played an irreplaceable role in Coronavirus Disease 2019 (COVID‐19) scientific research as well as medicine and vaccine research and development. With a high degree of academic acumen and a sense of national mission, the editorial team of *AMEM* took the lead in publishing the research results of the new coronary pneumonia model of the rhesus monkey in the first issue in March 2020. At present, this article has been cited 134 times, setting the highest citation rate since *AMEM* was published, significantly improving *AMEM*'s international dissemination power and also reflecting the social responsibility of the journal's expert team and manuscript authors in public health emergencies. The research results of the rhesus monkey’s new coronary pneumonia model by the research team of the editor‐in‐chief were successfully selected as the “Top Ten Progresses in Life Sciences in China” in 2020.

At the beginning of the new year, *AMEM* changed from a quarterly publication to a bimonthly publication. *AMEM* will continue to uphold its original aspirations, adapt to the needs of the times to accelerate the construction of international frontier hotspots columns, and enhance its international influence by collecting contributions from top international experts. We will focus on the current frontier hotspots of international basic medical research, such as cardiovascular and cerebrovascular diseases, neurological disease, natural drugs, etc., and publish the summaries and research articles related to them. At the same time, we will increase our online promotion efforts and develop together online and offline to promote the international dissemination and promotion of the results of basic medicine. High‐quality academic papers are the soul of the journal, while members of the editorial board are the guardians of the soul. In the face of new opportunities and challenges, *AMEM* has added more than 60 editorial members, covering basic medicine and clinical research as well as life science research. We warmly welcome more domestic and foreign experts with international perspectives to join us to promote the integration and cooperation in all aspects of basic medicine and simultaneously inject new forces into the development of experimental medicine.

We thank the executive editor and other experts of the editorial board for their hard work. *AMEM* looks forward to more exchanges and cooperation with domestic and international counterparts.

I deeply wish the journal a promising future in its fifth year and wish you all a happy new year!

